# ECG Signal De-noising and Baseline Wander Correction Based on CEEMDAN and Wavelet Threshold

**DOI:** 10.3390/s17122754

**Published:** 2017-11-28

**Authors:** Yang Xu, Mingzhang Luo, Tao Li, Gangbing Song

**Affiliations:** 1 Electronics & Information School, Yangtze University, Jingzhou 434023, China; xuyang@yangtzeu.edu.cn (Y.X.); ailin@yangtzeu.edu.cn (T.L.); 2 National Demonstration Center for Experimental Electrotechnics and Electronics Education, Yangtze University, Jingzhou 434023, China; 3 Department of Mechanical Engineering, University of Houston, Houston, TX 77004, USA

**Keywords:** electrocardiogram (ECG) signal, complete ensemble empirical mode decomposition with adaptive noise (CEEMDAN), wavelet threshold, random noise, de-noise, baseline wander

## Abstract

A novel electrocardiogram (ECG) signal de-noising and baseline wander correction method based on complete ensemble empirical mode decomposition with adaptive noise (CEEMDAN) and wavelet threshold is proposed. Although CEEMDAN is based on empirical mode decomposition (EMD), it represents a significant improvement of the original EMD by overcoming the mode-mixing problem. However, there has been no previous study on using CEEMDAN to de-noise ECG signals, to the authors’ best knowledge. In the proposed method, the original noisy ECG signal is decomposed into a series of intrinsic mode functions (IMFs) sorted from high to low frequency by CEEMDAN. Each IMF is then analyzed by the autocorrelation method to find out the first few high frequency IMFs containing random noise, and these IMFs should be de-noised by the wavelet threshold. The zero-crossing rate (ZCR) of all IMFs, including final residue, are computed, and the IMFs with ZCR less than a certain value are removed. Finally, the remaining IMFs are reconstructed to obtain the clean ECG signal. The proposed algorithm is validated through experiments using the MIT–BIH ECG databases, and the results show that the random noise in the ECG signal can be effectively suppressed, and at the same time the baseline wander can be corrected efficiently.

## 1. Introduction

**Figure 1:**
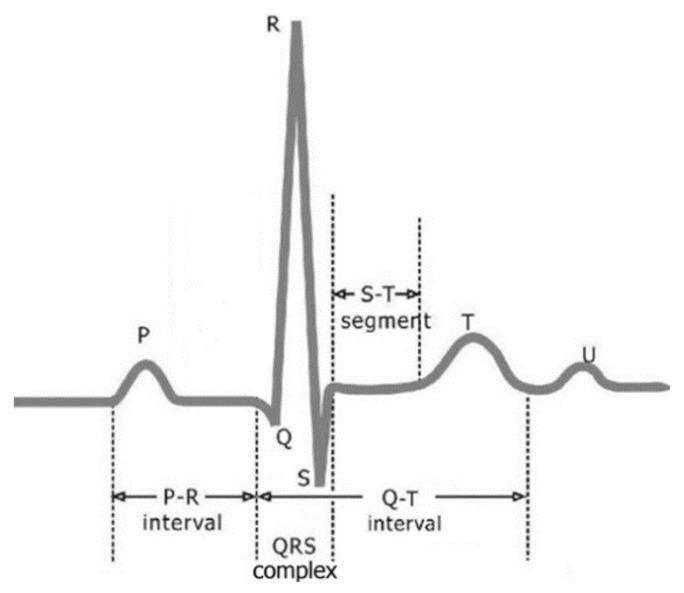
The electrocardiogram (ECG) of a heart in a normal sinus rhythm.

The electrocardiogram (ECG) has been widely used for the clinical diagnosis of heart disease. ECG is a weak, non-linear and non-stationary human physiological signal. The ECG in a normal sinus rhythm, as shown in Figure 1, consists of waveform components that indicate electrical events during one heartbeat. These waveforms are labeled P, Q, R, S, T and U. The P wave is the first deflection and is normally an upward waveform. It indicates the atrial depolarization. The QRS complex follows the P wave and it normally begins with a downward deflection, Q, then a larger upward deflection, R, and lastly a downward S wave. The QRS complex represents the depolarization of the right and left ventricles. The T wave is normally a modest upward waveform, representing ventricular repolarization. The U wave is hypothesized to be caused by the repolarization of the interventricular septum, and this wave component may not be observable. The PR interval is measured from the beginning of the P wave to the beginning of the QRS complex. This interval reflects the time the electrical impulse takes to travel from the sinus node through the atrioventricularnode. The ST segment is the line from the end of the QRS complex to the beginning of the T wave, and represents the period when the ventricles are depolarized. The QT interval represents the time of ventricular activity, including both depolarization and repolarization. It is measured from the beginning of the QRS complex to the end of the T wave [1]. The actual shape of the ECG waveform depends on the recording aspect (i.e., electrode position).

In the past few years, methods have been proposed to obtain basic medical characteristics from an ECG [2,3,4,5,6]. Laguna et al. [7] presented an automatic waveform boundaries detection algorithm for multiple lead ECG signals. Given these locations, features of clinical importance, such as each wave duration, may be readily obtained. Stridh and Sornmo [8] proposed a spatiotemporal approach to QRST cancellation in ECGs with atrial fibrillation, and this method compensates for morphological changes caused by variations in the electrical axis and uses linear transformations to combine multiple lead information. De Chazal et al. [9] developed a method for the automatic processing of the ECG signal for the classification of heartbeats. Rubel et al. [10] proposed a method of quantitative assessment of the extent and significance of serial ECG changes of the repolarization phase.

In practice, many adverse factors negatively impact the ECG signal during the data acquisition and transmission process, resulting in signal deviation and diagnostic inaccuracy. These adverse factors include various noises such as Gaussian noise, muscle artifacts, power-line interference, and baseline wander. To suppress noise and to obtain a clean ECG signal, many different algorithms are proposed [11,12,13]. The commonly used ECG signal de-noising methods include the morphological filtering method, the adaptive filtering method, the wavelet-based method, and the empirical mode decomposition (EMD) method. The morphological filtering method can achieve good effect when filtering the limit interference signal [14], however, a truncation error will be produced when filtering the high-frequency interference signal. The adaptive filtering (AF) method can automatically adjust the filter quality factor according to the noise characteristics of the signal. Agostinelli et al. [15] proposed a segmented beat modulation method for ECG estimation from noisy recordings. Ren et al. [16] introduced an improved adaptive algorithm for interference cancellation of ECG signals. Lim et al. [17] proposed an adaptive signal extraction method that uses discrete wavelet transformation coupled with adaptive parameters to address variated ECG signals due to varying heartrates. In addition, the adaptive filtering de-noising effect is good, and the waveform cannot be easily distorted; however, the computation load is quite heavy with AF.

Wavelet transform is a kind of mature method for signal processing [18]. With the characteristics of different scales and low entropy, it has great advantages in non-stationary signal processing. In the wavelet threshold de-noising method proposed by Donoho [19], combined with the existing orthogonal wavelet decomposition and reconstruction algorithm, a complete set of threshold de-noising strategies have been developed, and they can be used for suppressing random noise. Yi et al. [20] employed an improved wavelet threshold technique for noise smoothing, and achieved a good effect. Singh and Tiwari [21] presented a selection procedure of mother wavelet basis functions applied to de-noising of the ECG signal in the wavelet domain while retaining the signal peaks close to their full amplitude. The obtained wavelet-based de-noised ECG signals retain the necessary diagnostics information contained in the original ECG signal. Liu et al. [22] proposed an improved wavelet threshold algorithm for ECG de-noising. Das et al. [23] proposed a wavelet based de-noising technique by thresholding the less significant wavelet coefficients. Here, the threshold is based on the probability of the wavelet coefficients at a particular sub-band. The data used for testing purposes is taken from MIT–BIH Database. Chen and Chen [24] developed a hardware design and implemented a wavelet de-noising procedure for medical signal preprocessing. The wavelet threshold method can effectively remove the noise interference in ECG signals, but the choice of threshold is a troublesome process, and has a direct impact on the noise-filtering effect.

The empirical mode decomposition (EMD) method has many advantages for non-stationary signal analysis [25,26,27], and the EMD can be used for ECG noise reduction. Blanco-Velasco et al. [28] proposed an EMD-based algorithm to remove the baseline wander and high-frequency noise of ECGs. Singh and Sunkaria [29] developed an ECG signal de-noising method based on the empirical mode decomposition and the moving average filter. The proposed method is an enhancement of the existing EMD-based de-noising algorithms. Kabir and Shahnaz [30] presented an ECG de-noising approach based on noise-reduction algorithms in EMD and discrete wavelet transform. Unlike the conventional EMD-based ECG de-noising approaches that neglect a number of initial intrinsic mode functions (IMFs) containing the QRS complex as well as noise, the new approach performs windowing in the EMD domain to reduce the noise from the initial IMFs instead of discarding them completely, thus preserving the QRS complex and yielding a relatively cleaner ECG signal.

The major disadvantage of EMD is the so-called mode-mixing effect. The mode mixing indicates that oscillations of different time scales coexist in a given intrinsic mode function (IMF), or that oscillations with the same time scale have been assigned to different IMFs. To overcome these problems, Wu and Huang [31] proposed an ensemble empirical mode decomposition (EEMD) algorithm, which performs the decomposition over an ensemble of noisy copies of the original signal, obtaining the final results by averaging. The addition of white Gaussian noise reduces the mode mixing by populating the whole time–frequency space, taking advantage of the dyadic filter bank behavior of the EMD. Thus, more regular modes are obtained, with similar scales for the entire time span. Zhao et al. [32] developed a human ECG identification system based on ensemble empirical mode decomposition. Chang [33] proposed a noise-filtering algorithm based on EEMD to remove artifacts in ECG signals. Ye et al. [34] studied the de-noising method of ECG signals based on the EEMD and improved the wavelet threshold. Chang [35] studied EEMD for high-frequency ECG noise reduction. Even if EEMD has shown to be useful in a wide range of applications, it also creates new difficulties. The white Gaussian noise added by EEMD cannot be completely canceled after a finite average, resulting in a reconstruction error. Although with the increase of the average time, the reconstruction error can be reduced, the computational cost is greatly increased. To improve EEMD, Torres et al. [36] proposed the complete ensemble empirical mode decomposition with adaptive noise (CEEMDAN) algorithm. By adding adaptive white Gaussian noise in every stage of EMD, CEEMDAN obtains every IMF mode through calculating the unique residue. With the CEEMDAN, the decomposition process can effectively overcome the mode-mixing problem, and the reconstruction error is almost zero, with greatly reduced computational cost. Therefore, the CEEMDAN represents a significant improvement on the original EMD. However, there has been no previous study of using CEEMDAN to de-noise ECG signals, to the authors’ best knowledge.

In this paper, a novel ECG signal de-noising algorithm based on CEEMDAN and wavelet threshold is proposed. The noisy ECG signal is decomposed into a series of IMFs sorted from high to low frequency, and each IMF is analyzed by the autocorrelation method to find out the first few IMFs containing random noise [37]. These IMFs should first be de-noised by the wavelet threshold. Then, the zero crossing rate (ZCR) of all IMFs, including the final residue, is computed. Removing the IMFs with ZCR less than a certain value allows for correction of the baseline wander in the ECG signal [38]. After the remaining IMFs are reconstructed, the clean ECG signal is obtained. This methodology is validated through experiments on the MIT–BIH ECG databases, and results show that the random noise in the ECG signal can be effectively suppressed and the baseline wander can be corrected efficiently as well.

## 2. Basic Principle

### 2.1. EMD, EEMD, CEEMDAN Algorithm

The EMD decomposes a signal *s*(*t*) into a number of intrinsic mode functions (IMFs) or modes. To be considered as an IMF, the following two conditions must be satisfied: (1) the total extreme points and zero crossings should be equal, or at most differ by one; and (2) at any time, the mean of upper and lower envelope formed by the local extreme points is zero.

The procedure of the EMD algorithm is described as follows:

Step 1. Find all extreme points (both minima and maxima) in the test signal *s*(*t*).
Step 2. Use the cubic spline interpolation between maxima (minima) to obtain the upper (lower) envelope *u*_0_(*t*) (*d*_0_(*t*)).
Step 3. Compute the mean envelope,
  (1) $$m_{0}(t)=\frac{1}{2}(u_{0}(t)+d_{0}(t))$$
Step 4. Subtract the mean envelope from *s*(*t*) to obtain,
  (2) $$h_{1}(t)=s(t)-m_{0}(t)$$
Step 5. If *h*_1_(*t*) follows the criteria of the IMF, then it is the *IMF*_1_, otherwise *h*_1_(*t*) is considered as the data of the sifting process, and repeat steps 1 to 4. Thus a new function *h*_11_(*t*) is obtained. This process will be repeated until either *h*_1__k_(*t*) follows the criteria of IMF or a certain termination condition (generally standard deviation criteria) is met.
Step 6. Subtract the *IMF*_1_ from *s*(*t*) to obtain the residue,
  (3) $$r_{1}(t)=s(t)-IMF_{1}$$
Step 7. Treat the residual $r_{1}(t)$ as a new signal, repeat steps 1 to 6, and other IMFs such as *IMF*_2_, *IMF*_3_, …, *IMF_N_* can be obtained, until $r_{N}(t)$ becomes either a constant, a monotonic slope, or a function with only one extremum. After the EMD decomposition, the original signal *s*(*t*) can be expressed as,
  (4) $$s(t)=\sum_{i=1}^{N}IMF_{i}+r_{N}(t)$$
  where *N* is the number of IMFs, and $r_{N}(t)$ is the final residue.

The EMD method has many advantages for non-stationary signal analysis, and is suitable for ECG de-noising. The major disadvantage of EMD is the so-called mode-mixing effect. By adding finite Gaussian white noise, the EEMD method largely eliminates the mode-mixing of the EMD.

The procedure of the EEMD algorithm is described as follows:

Step 1. The Gaussian white noise with zero mean and unit variance $n_{i}(t)$ is added to the original signal s(t), to obtain a new signal,
  (5) $$s_{i}(t)=s(t)+n_{i}(t)$$
Step 2. For every *i* = 1, …, *I*, decompose each $s_{i}(t)$ by EMD, to obtain *IMF_ik_*, where *k* = 1, …, *N* denote the number of IMFs.
Step 3. Average *IMF_ik_*, to obtain EEMD mode,
  (6) $${\bar{IMF}}_{k}=\frac{1}{I}\sum_{i=1}^{I}IMF_{ik}$$

Even though EEMD eliminated the mode mixing, the Gaussian white noise added by the EEMD cannot be completely canceled after a finite average, resulting in a reconstruction error. The CEEMDAN is an improvement on EEMD. With CEEMDAN, the decomposition process can effectively overcome the mode-mixing problem, and the reconstruction error is almost zero, with greatly reduced computational cost.

The procedure of the CEEMDAN algorithm is described as follows:

Define *E_j_*(·) as the operator that produces the *j-*th mode obtained by EMD, and let $n_{i}(t)$ be a realization of zero mean unit variance white noise. If *s*(*t*) is the target signal, the CEEMDAN algorithm steps are as follows:

Step 1. For every *i* = 1,…, *I*, decompose each $s_{i}(t)=s(t)+\varepsilon_{0}n_{i}(t)$ by EMD, until its first mode, and define the first CEEMDAN mode as,
  (7) $${\tilde{IMF}}_{1}=\frac{1}{I}\sum_{i=1}^{I}IMF_{i1}$$
Step 2. At the first stage (*j* = 1), calculate the first residue,
  (8) $$r_{1}(t)=s(t)-{\tilde{IMF}}_{1}$$
Step 3. For every *i* = 1, …, *I*, decompose each $r_{1}(t)+\varepsilon_{1}E_{1}(n_{i}(t))$ by EMD, until its first mode, and define the second CEEMDAN mode as,
  (9) $${\tilde{IMF}}_{2}=\frac{1}{I}\sum_{i=1}^{I}E_{1}(r_{1}(t)+\varepsilon_{1}E_{1}(n_{i}(t)))$$
Step 4. For *j* = 2, 3, …, *N*, calculate the *j-*th residue,
  (10) $$r_{j}(t)=r_{j-1}(t)-{\tilde{IMF}}_{j}$$
Step 5. For every *i* = 1, …, *I*, decompose each $r_{j}(t)+\varepsilon_{j}E_{j}(n_{i}(t))$ by EMD, until its first mode, and define the (*j* + 1)-th CEEMDAN mode as,
  (11) $${\tilde{IMF}}_{j+1}=\frac{1}{I}\sum_{i=1}^{I}E_{1}(r_{j}(t)+\varepsilon_{j}E_{j}(n_{i}(t)))$$
Step 6. Go to step 4 for the next *j*.

Repeat steps 4 to 6 until the obtained residue can no longer be further decomposed by the EMD, because either it satisfies IMF criteria or it has been less than the three local extrema. The final residue satisfies,

(12) $$r_{N}(t)=s(t)-\sum_{j=1}^{N}\tilde{IMF_{j}}$$

with *N* being the total number of modes. Therefore, the target signal can be expressed as,

(13) $$s(t)=\sum_{j=1}^{N}\tilde{IMF_{j}}+r_{N}(t)$$

which ensures the completeness property of the CEEMDAN decomposition, thus providing an exact reconstruction of the original data. The final number of modes is determined only by the data and the stopping criterion. The coefficient *ε* allows the selection of the SNR at each stage.

### 2.2. Improved Wavelet Threshold Function

The key to de-noising based on wavelet threshold is the selection of the threshold function. The hard and soft threshold functions proposed by Donoho are widely used in practice. Combining the different characteristics of hard and soft threshold functions, this paper adopts an improved threshold function [39] for estimation of wavelet coefficients,

(14) $${\tilde{\omega }}_{j,k}=\left\{ \begin{array}{ll} \mathrm{sgn}(\omega_{j,k})[|\omega_{j,k}|-t^{c\cdot (\lambda_{j}-|\omega_{j,k}|)}\lambda_{j}], & |\omega_{j,k}|\geq \lambda_{j}\\ 0, & |\omega_{j,k}|<\lambda_{j} \end{array}\right.$$

where $\omega_{j,k}$ denotes the *k*-th wavelet coefficients under *j*-th scale decomposition, ${\tilde{\omega }}_{j,k}$ denotes the estimated wavelet coefficients.$t=\sqrt{2\ln N}$, *N* is the signal length. $\lambda_{j}$ is the threshold, and $\lambda_{j}=\sigma_{j}t=\sigma_{j}\sqrt{2\ln N}$ with $\sigma_{j}$ being the standard deviation of noise.

When $\left|\omega_{j,k}\right|$ is close to the threshold $\lambda_{j}$, ${\tilde{\omega }}_{j,k}\approx \mathrm{sgn}(\omega_{j,k})[|\omega_{j,k}|-\lambda_{j}]$, and Equation (14) is similar to the soft threshold function. When c→0, Equation (14) is the same as soft threshold function. When c→∞, Equation (14) is the same as the hard threshold function. 

The improved threshold function is a compromise between the hard threshold and soft threshold functions. Using the improved threshold function, the reconstructed signal can preserve the original signal’s characteristics.

## 3. ECG Signal De-Noising Procedure

### 3.1. Flowchart of ECG Signal De-Noising

**Figure 2:**
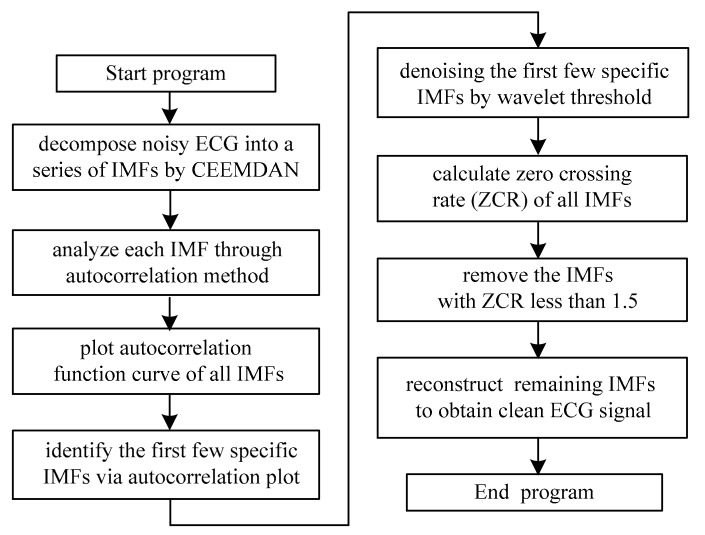
The flowchart of ECG signal de-noising.

The ECG signal de-noising based on the CEEMDAN and wavelet threshold undergoes the following procedure. Decompose the noisy ECG signal into a series of IMFs sorted from high to low frequency by CEEMDAN, and the first few IMFs contain random noise. To find out the first few specific IMFs, each IMF is analyzed through the autocorrelation method. Once the IMF containing the random noise is identified, it should be de-noised by the wavelet threshold. Then, the zero crossing rates (ZCR) of all IMFs including the final residue are calculated, and remove the IMFs with ZCR less than 1.5. Finally, reconstruct the remaining IMFs so as to obtain the clean ECG signal. The flowchart of ECG signal de-noising is shown in Figure 2.

### 3.2. Random Noise Suppression in the ECG Signal

The noisy ECG signal is decomposed into a series of IMFs sorted from high to low frequency by CEEMDAN. The first few IMFs contain high-frequency random noise. Random noise suppression by the EMD is in general carried out by partial signal reconstruction, and the first few high frequency IMFs are removed. This method will introduce ECG signal distortion due to some useful information such as the QRS complex that may be contained in the first few high-frequency IMFs. On the other hand, the exact number of the first few high-frequency IMFs containing random noise is hard to determine. This paper employs autocorrelation analysis to find out the first few high-frequency IMFs. Instead of removing these specific high-frequency IMFs, they should be de-noised with the wavelet threshold so as to suppress the random noise. This method will not result in an ECG signal distortion.

The autocorrelation function is used to measure the similarity between signal x(t) and signal x(t+τ); the normalized autocorrelation function is defined as,

(15) $$R_{x}(\tau )=\frac{E[x(t)\cdot x(t+\tau )]}{E[x(t)\cdot x(t)]}$$

where $R_{x}(\tau )$ denotes the normalized autocorrelation function, and *E*(·) represents the mean value.

The autocorrelation function plot of pure random noise is a sharp pulse, however, the plot of a noisy ECG signal has a certain width. The noisy ECG signal is decomposed into a series of IMFs by CEEMDAN, when there is more random noise in an IMF, and the central part of the autocorrelation function plot narrows. Therefore, the first few specific IMFs containing random noise can be determined according to the autocorrelation function plots.

**Figure 3:**
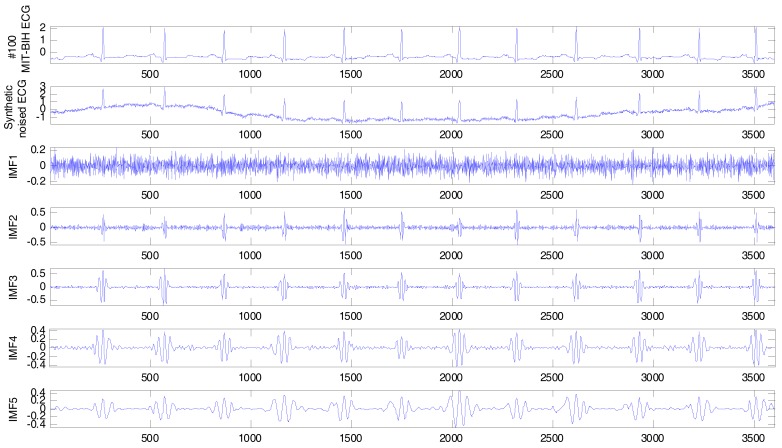

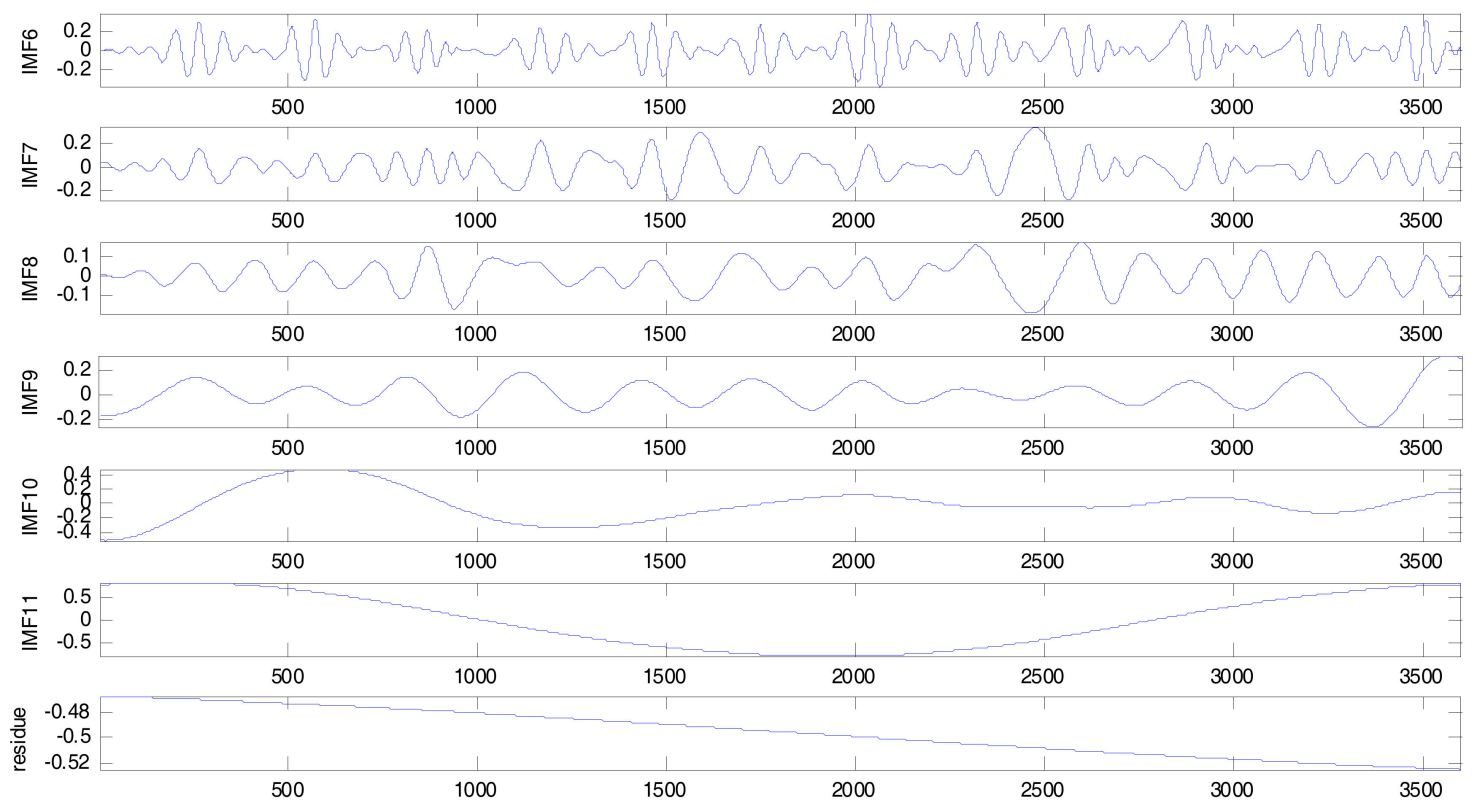
Synthetic noisy ECG signal and its intrinsic mode functions (IMFs) decomposed by complete ensemble empirical mode decomposition with adaptive noise (CEEMDAN).

Here, we select the No. 100 ECG record from the MIT–BIH arrhythmia database for the experiment. The sampling frequency is 360 Hz. The sampling duration is 10 s. To synthesize a noisy ECG signal, 15 dB Gaussian white noise and the baseline wander signal from the MIT–BIH noise stress test database are added to the No. 100 ECG signal. The synthetic noisy ECG signal is decomposed into 11 IMFs and 1 residue component by CEEMDAN, as shown in Figure 3.

**Figure 4:**
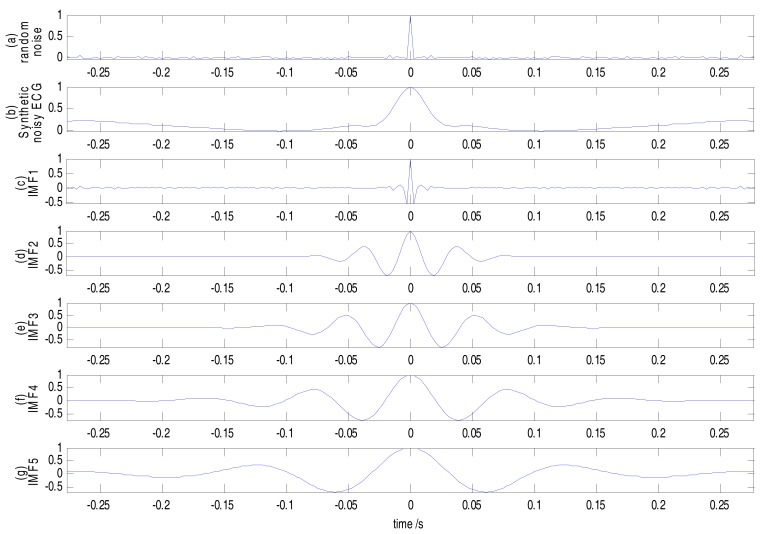
Partial autocorrelation function plots of the synthetic noisy ECG signal.

The partial corresponding autocorrelation function plots are as shown in Figure 4. It can be seen from Figure 4b that the width of the central part of the synthetic noisy ECG autocorrelation function plot is about 50 ms. Only in Figure 4c–e (IMF1~IMF3) is the width of the central part of autocorrelation function plots less than 50 ms, and this illustrates IMF1~IMF3 containing much more random noise than the other IMFs. Therefore, only the first three IMFs (IMF1~IMF3) should be chosen for de-noising by the wavelet threshold to suppress random noise.

### 3.3. ECG Baseline Wander Correction

The ECG baseline wander is a slow-changing signal, and its frequency is less than 1.5 Hz. The baseline wander signal is decomposed into the final IMFs (including the final residue) by the CEEMDAN. The baseline wander can be corrected if the specific final IMFs are removed from the noisy ECG signal. Now the only issue is how to determine the specific final IMFs. Here, we employ the zero-crossing rate (ZCR) in order to do so. The ZCR is often used to extract the signal feature and has a relationship with frequency. Average ZCR can be used to estimate roughly the frequency of an IMF. As shown in Figure 3, the synthetic noisy ECG signal is decomposed into 11 IMFs and 1 residue component. The ZCRs of all the IMFs, including the residue component, are calculated and as listed in Table 1. We can tell the last three ZCRs are less than 1.5, so the IMF10~IMF11 as well as the residue component should be removed. The IMFs with frequencies less than 1.5 Hz are considered as the baseline wander signals. Then, after the remaining IMFs are reconstructed, the ECG baseline wander correction can be achieved and the clean ECG signal is obtained.

## 4. ECG Signal De-Noising Results

### 4.1. Synthetic Noisy ECG Signal De-Noising Results

**Figure 5:**
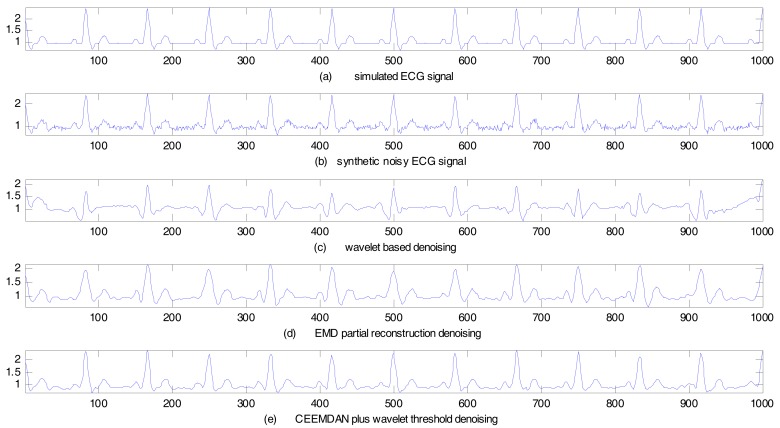
Synthetic noisy ECG (simulated ECG) signal de-noising results.

To obtain the noise-free ECG signal, we adopt the morphological modeling method [40] to simulate ECG waveforms. With this approach, the significant features of ECG, such as P, Q, R, S, and T waves, the duration of each wave, and certain time intervals, can be simulated. The ECG signal generated by the simulator is noise-free. To synthesize a noisy ECG signal, 15 dB Gaussian white noise is added to the simulated ECG signal. The synthetic noisy ECG signal is then de-noised by the proposed CEEMDAN plus wavelet threshold method. For the purpose of comparison, both the wavelet-based method and the EMD partial reconstruction method are applied. The de-noising results are shown in Figure 5.

Figure 5a shows the simulated ECG signal, Figure 5b–e, plot the synthetic noisy ECG signal, the wavelet-based de-noising result, the EMD partial reconstruction de-noising result, and the proposed CEEMDAN plus wavelet threshold de-noising result, respectively. In the wavelet-based method, a 6-level discrete wavelet transform with the Symlet wavelet of order 7 (Sym7) is used for de-noising. The Symlet family wavelets are popular for signal de-noising because of their energy concentration at low frequency. In the EMD partial reconstruction method, the noisy ECG signal is decomposed into a series of IMFs to remove the first two high-frequency IMFs, and then the remaining IMFs are reconstructed to obtain de-noising results.

To illustrate the advantages of the proposed algorithm quantitatively, signal-to-error ratio (SER) and mean square error (MSE) are used as the performance indices of de-noising. SER defines the signal energy with respect to the energy of the error. MSE defines the energy of the error signal in the de-noising process.

The formulas of SER and MSE are, respectively, given as,

(16) $$SER=10{\mathrm{lg}}_{10}\left\{\frac{\sum_{i=1}^{N}s_{i}^{2}(t)}{\sum_{i=1}^{N}{\left(s_{i}(t)-{\hat{s}}_{i}(t)\right)}^{2}}\right\}$$

(17) $$MSE=\frac{1}{N}\sum_{i=1}^{N}{\left(s_{i}(t)-{\hat{s}}_{i}(t)\right)}^{2}$$

where $s_{i}(t)$ is the original ECG signal, ${\hat{s}}_{i}(t)$ is the de-noised ECG signal, and *N* is the signal length.

Performance indices computed under different noise intensity are as listed in Table 2. Table 2 clearly shows that the proposed method has the highest SER and lowest MSE, which demonstrates the superiority of the proposed method over other methods.

**Figure 6:**
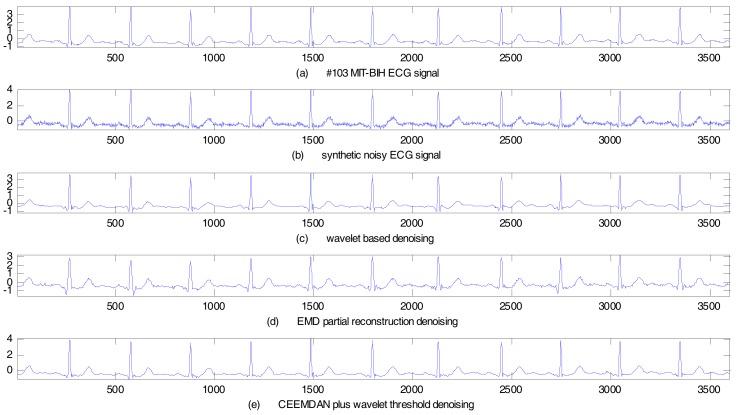
Synthetic noisy ECG signal de-noising results (No. 103 MIT-BIH ECG).

We also select the No. 103 ECG record from the MIT-BIH arrhythmia database for experiment. The sampling frequency is 360 Hz. The sampling duration is 10 s. The synthetic noisy ECG signal is achieved by adding 15 dB Gauss white noise. The wavelet based method, the EMD partial reconstruction method and the proposed CEEMADN plus wavelet threshold method are applied to de-noise the synthetic noisy ECG signal, respectively. The de-noising results are as shown in Figure 6. Performance indices computed under different noise intensity are as listed in Table 3. Once again, the table clearly shows that the proposed method has the highest SER and lowest MSE, which demonstrates the superiority of the proposed method over other methods.

**Figure 7:**
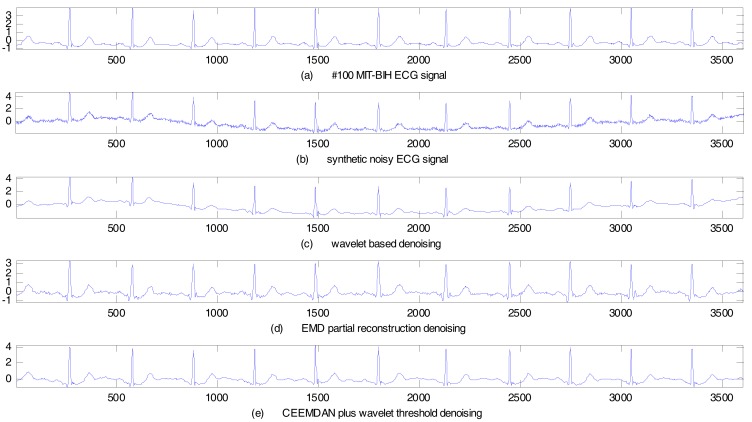
Synthetic noisy ECG with baseline wander de-noising results (No. 100 MIT–BIH ECG).

We now focus on the case when both random noise and baseline wander appear in the ECG signal. We selected the No. 100 ECG record from the MIT–BIH arrhythmia database for the experiment. The sampling frequency was 360 Hz. The sampling duration was 10 s. To synthesize a noisy ECG signal, 15 dB Gauss white noise and the baseline wander signal from the MIT–BIH noise stress test database were added to the ECG signal. The synthetic noisy ECG signal was de-noised by the same three kinds of method described above. The de-noising results are shown in Figure 7. Performance indices computed under different noise intensities are as listed in Table 4. As with the previous cases, Table 4 clearly shows that the proposed method has the highest SER and lowest MSE, which demonstrates the superiority of the proposed method over other methods.

In summary, as the objective of this study was to de-noise the ECG signals and to remove the baseline wonder, the SER is a relevant performance index to quantify the efficacy of reducing noise. MSE tracks the accuracy of the de-noising technique to estimate the original signal. Hence, a larger SER value denotes a better de-noising effect, and a lower MSE value denotes a better estimation of the original signal and a better preservation of signal details. As can be observed from Table 2, Table 3 and Table 4, the proposed CEEMDAN plus wavelet threshold method attains a greater SER value as well as a lower MSE value, as compared to the other two methods with respect to the given noise intensity, in both cases of the simulated ECG signal and the MIT–BIH arrhythmia database. This illustrates that the proposed method has a better de-noising effect. The EMD partial reconstruction method attains a lower SER (larger MSE) than that of the proposed method. Although the EMD method can reduce the noise in the ECG signal, the de-noising effect is not good enough due to the drawback of mode-mixing. The wavelet based method attains the worst SER and MSE values in the given noise-intensity cases. On the other hand, we can see the behavior of the wavelet-based method in Figure 7c, where only the random noise is smoothened, however it is unable to remove the baseline wander signal components.

### 4.2. Real ECG Signal De-Noising Results

**Figure 8:**
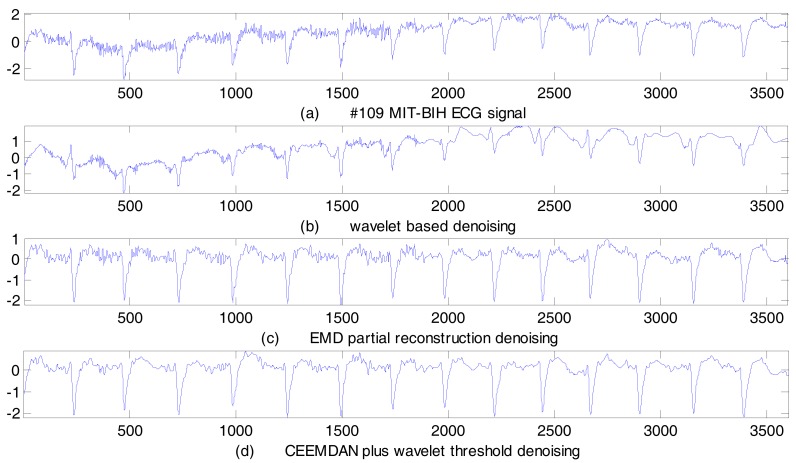
No. 109 MIT–BIH ECG signal de-noising results.

**Figure 9:**
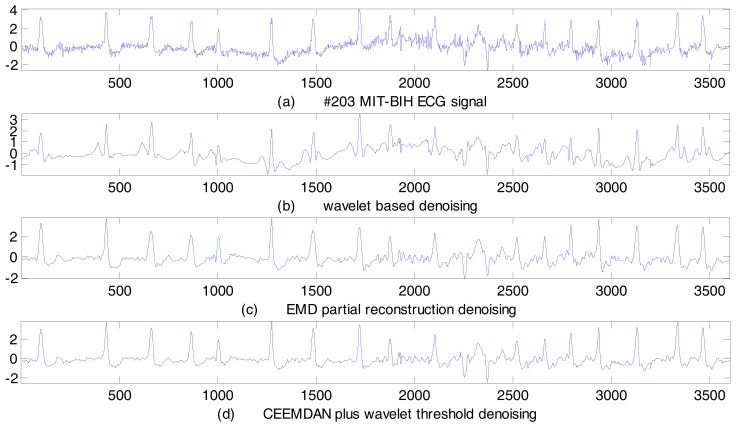
No. 203 MIT–BIH ECG signal de-noising results.

We selected the No. 109 and No. 203 ECG records from the MIT–BIH arrhythmia database for real signal de-noising. These two records were subject to severe random noise and baseline wander interference. We employed the proposed CEEMADN plus wavelet threshold method to de-noise these two real ECG signals. As a comparison, both the wavelet-based method and the EMD partial reconstruction method were also employed, and the de-noising results are shown in Figure 8 and Figure 9. Performance indices are listed in Table 5.

As can be observed from Table 5, with the wavelet-based de-noising method, both the SER and the MSE values are not as good as the other two de-noising methods. From Figure 8b and Figure 9b, we can tell that only the random noise have been smoothened, but the baseline wander signal cannot be removed. This shows that the wavelet-based de-noising method has some limitations in real ECG signal de-noising cases. With the EMD partial reconstruction method, the SER and the MSE values are better than those of the wavelet-based method, and it can achieve a better de-noising result. From Figure 8c and Figure 9c, we can see not only that the random noise is suppressed, but also that the baseline wander is corrected. Even so, this method will introduce ECG signal distortion owing to the EMD mode-mixing. Among the three de-noising methods, the proposed CEEMDAN plus wavelet threshold method is the best. In both the No. 109 ECG record and the No. 203 ECG record situations, the SER is the largest, and the MSE is the lowest. The de-noising results are very clear from Figure 8d and Figure 9d, the random noise is obviously reduced, and the baseline wander is corrected effectively. These results further demonstrate that the proposed method is suitable for real ECG signal de-noising in different cases.

## 5. Conclusions

In this paper, a novel ECG signal de-noising and baseline wander correction algorithm based on CEEMDAN and the wavelet threshold is proposed. The noisy ECG signal is decomposed into a series of IMFs. The first few IMFs containing random noise are determined by the autocorrelation analysis and are de-noised by the wavelet threshold. The last few IMFs with ZCR less than 1.5 are removed for baseline wander correction. Finally, the remaining IMFs are reconstructed to obtain the clean ECG signal. The proposed algorithm is validated through experiments on both the simulated synthetic noisy ECG signal and the MIT–BIH ECG databases. The results show that the random noise can be suppressed effectively and the baseline wander can be corrected efficiently. As a new signal-processing method, CEEMDAN has advantages in dealing with non-linear and non-stationary signals. Although CEEMDAN is based on empirical mode decomposition (EMD), it represents a significant improvement on the original EMD by overcoming the mode-mixing problem. This advanced technique will be beneficial for future computer-based automated diagnostic systems. We use the SER and the MSE as quality estimators for de-noising, as described earlier. Recently, a new type of weighted diagnostic distortion (WDD) estimating method has been introduced [40]. Although WDD has the disadvantage of requiring heavy computation, it correlates better with the real quality of the tested ECG signal, and we will try to use this kind of quality estimator in our future work. In this paper, we focus only on random noise reduction and baseline wander correction; however this is not the limit of our work in this area. We will continue to improve the ECG signal de-noising method. Our future work will involve WDD and muscular noise cancellation, which is another challenge.

## Figures and Tables

**Table 1:** Zero-crossing rates (ZCRs) of all the IMFs for the No. 100 ECG signal.

IMF	IMF1	IMF2	IMF3	IMF4	IMF5	IMF6	IMF7	IMF8	IMF9	IMF10	IMF11	Residue
ZCR	246.6	119.2	61.4	33.5	18.7	11.4	6.6	4.1	2.3	0.7	0.2	0.0

**Table 2:** Performance indices under different noise intensity (simulated ECG).

Noise Intensity	Index	Wavelet Based	EMD Partial Reconstruction	CEEMDAN Plus Wavelet Threshold
5 dB	SER	0.8751	6.8711	18.3150
	MSE	0.0452	0.0218	0.0016
10 dB	SER	0.5978	5.9251	17.0436
	MSE	0.0466	0.0271	0.0021
15 dB	SER	0.4232	4.8161	12.5823
	MSE	0.0492	0.0295	0.0056
20 dB	SER	0.2435	3.9363	10.4129
	MSE	0.0709	0.0638	0.0097

**Table 3:** Performance indices under different noise intensity (No. 103 MIT-BIH ECG).

Noise Intensity	Index	Wavelet-Based	EMD Partial Reconstruction	CEEMDAN Plus Wavelet Threshold
5 dB	SER	14.7072	16.2526	27.8397
	MSE	0.0201	0.0113	0.0006
10 dB	SER	13.1222	15.6894	25.3689
	MSE	0.0210	0.0129	0.0011
15 dB	SER	11.7577	13.9286	20.4182
	MSE	0.0316	0.0154	0.0035
20 dB	SER	10.6856	11.9397	16.1626
	MSE	0.0405	0.0217	0.0095

**Table 4:** Performance indices under different noise intensity (No. 100 MIT–BIH ECG).

Noise Intensity	Index	Wavelet-Based	EMD Partial Reconstruction	CEEMDAN Plus Wavelet Threshold
5 dB	SER	14.9580	17.9656	26.6223
	MSE	0.0213	0.0096	0.0010
10 dB	SER	12.8418	16.1767	24.8403
	MSE	0.0235	0.0105	0.0016
15 dB	SER	11.3410	14.8742	21.6968
	MSE	0.0380	0.0124	0.0041
20 dB	SER	10.3478	13.7996	17.8879
	MSE	0.0438	0.0198	0.0097

**Table 5:** Real ECG signal de-noising performance indices data.

MIT–BIH ECG Record	Index	Wavelet-Based	EMD Partial Reconstruction	CEEMDAN Plus Wavelet Threshold
No. 109	SER	8.2816	13.6221	19.7232
	MSE	0.0560	0.0317	0.0196
No. 203	SER	12.0928	16.4504	21.7684
	MSE	0.0510	0.0409	0.0284

## References

[1] Brady W.J., Hudson K.B., Naples R., Sudhir A., Mitchell S.H., Ferguson J.D. (2013). The ECG in Prehospital Emergency Care.

[2] Raka A.G., Naik G.R., Chai R. (2017). Computational Algorithms Underlying the Time-Based Detection of Sudden Cardiac Arrest via Electrocardiographic Markers. Appl. Sci..

[3] Peng Y., Wang X., Guo L., Wang Y., Deng Q. (2017). An Efficient Network Coding-Based Fault-Tolerant Mechanism in WBAN for Smart Healthcare Monitoring Systems. Appl. Sci..

[4] Weder M., Hegemann D., Amberg M., Hess M., Boesel L.F., Abächerli R., Meyer V.R., Rossi R.M. (2015). Embroidered Electrode with Silver/Titanium Coating for Long-Term ECG Monitoring. Sensors.

[5] Abtahi F., Snäll J., Aslamy B., Abtahi S., Seoane F., Lindecrantz K. (2015). Biosignal PI, an Affordable Open-Source ECG and Respiration Measurement System. Sensors.

[6] Chen Y.-H., de Beeck M.O., Vanderheyden L., Carrette E., Mihajlović V., Vanstreels K., Grundlehner B., Gadeyne S., Boon P., Van Hoof C. (2014). Soft, Comfortable Polymer Dry Electrodes for High Quality ECG and EEG Recording. Sensors.

[7] Laguna P., Jan R., Caminal P. (1994). Automatic detection of wave boundaries in multilead ECG signals: Validation with the cse database. Comput. Biomed. Res..

[8] Stridh M., Sornmo L. Spatiotemporal QRST cancellation techniques for analysis of atrial fibrillation: Methods and performance. Proceedings of the Computers in Cardiology.

[9] De Chazal P., O’Dwyer M., Reilly R.B. (2004). Automatic classification of heartbeats using ECG morphology and heartbeat interval features. IEEE Trans. Bio-Med. Eng..

[10] Rubel P., Fayn J., Mohsen N., Girard P. (1988). New methods of quantitative assessment of the extent and significance of serial ECG changes of the repolarization phase. J. Electrocardiol..

[11] Guo X., Shen C., Chen L. (2017). Deep Fault Recognizer: An Integrated Model to De-noise and Extract Features for Fault Diagnosis in Rotating Machinery. Appl. Sci..

[12] Al-Tmeme A., Woo W.L., Dlay S.S., Gao B. (2017). Underdetermined convolutive source separation using gem-mu with variational approximated optimum model order nmf2d. IEEE/ACM Trans. Audio Speech Lang. Process..

[13] Al-Nima R.R.O., Abdullah M.A.M., Al-Kaltakchi M.T.S., Dlay S.S., Woo W.L., Chambers J.A. (2017). Finger texture biometric verification exploiting multi-scale sobel angles local binary pattern features and score-based fusion. Dig. Signal Process..

[14] Wang A.D., Liu L., Wei Q. (2012). An adaptive morphologic filter applied to ECG de-noising and extraction of r peak at real-time. AASRI Procedia.

[15] Agostinelli A., Sbrollini A., Giuliani C., Fioretti S., Nardo F.D., Burattini L. (2016). Segmented beat modulation method for electrocardiogram estimation from noisy recordings. Med. Eng. Phys..

[16] Ren A., Du Z., Li J., Hu F., Yang X., Abbas H. (2017). Adaptive Interference Cancellation of ECG Signals. Sensors.

[17] Lim C.L.P., Woo W.L., Dlay S.S. Enhanced wavelet transformation for feature extraction in highly variated ECG signal. Proceedings of the 2nd IET International Conference on Intelligent Signal Processing 2015 (ISP).

[18] Mallat S. (1999). A Wavelet Tour of Signal Processing. A Wavelet Tour of Signal Processing.

[19] Donoho D.L. (2002). De-noising by soft-thresholding. IEEE Trans. Inf. Theory.

[20] Yi T.-H., Li H.-N., Zhao X.-Y. (2012). Noise Smoothing for Structural Vibration Test Signals Using an Improved Wavelet Thresholding Technique. Sensors.

[21] Singh B.N., Tiwari A.K. (2006). Optimal selection of wavelet basis function applied to ECG signal denoising. Dig. Signal Process..

[22] Liu X., Qiao L., Yang J., Dong B., Wang H. (2014). An improved wavelet threshold algorithm for ECG denoising. J. Biomed. Eng..

[23] Das A., Nirmala S.R., Medhi J.P. ECG denoising based on probability of wavelet coefficients. Proceedings of the 2015 International Symposium on Advanced Computing and Communication (ISACC).

[24] Chen S.W., Chen Y.H. (2015). Hardware design and implementation of a wavelet de-noising procedure for medical signal preprocessing. Sensors.

[25] Xu J., Wang Z., Tan C., Si L., Liu X. (2017). A novel de-noising method for an acoustic-based system through empirical mode decomposition and an improved fruit fly optimization algorithm. Appl. Sci..

[26] Lv Y., Yuan R., Song G. (2016). Multivariate empirical mode decomposition and its application to fault diagnosis of rolling bearing. Mech. Syst. Signal Process..

[27] Zhao X., Li M., Song G., Xu J. (2010). Hierarchical ensemble-based data fusion for structural health monitoring. Smart Mater. Struct..

[28] Blanco-Velasco M., Weng B., Barner K.E. (2008). ECG signal de-noising and baseline wander correction based on the empirical mode decomposition. Comput. Biol. Med..

[29] Singh O., Sunkaria R.K. (2013). Ecg signal de-noising based on empirical mode decomposition and moving average filter. Int. J. Med. Eng. Inf..

[30] Kabir M.A., Shahnaz C. (2012). Denoising of ECG signals based on noise reduction algorithms in EMD and wavelet domains. Biomed. Signal Process. Control.

[31] Wu Z.H., Huang N.E. (2009). Ensemble empirical mode decomposition: A noise-assisted data analysis method. Adv. Adapt. Data Anal..

[32] Zhao Z., Yang L., Chen D., Luo Y. (2013). A Human ECG Identification System Based on Ensemble Empirical Mode Decomposition. Sensors.

[33] Chang K.-M. (2010). Arrhythmia ECG Noise Reduction by Ensemble Empirical Mode Decomposition. Sensors.

[34] Ye L.L., Yang D., Wang X. (2014). Research on ECG De-noising Method Based on Ensemble Empirical Mode Decomposition and Wavelet Transform Using Improved Threshold Function. J. Biomed. Eng..

[35] Chang K.M. (2010). Ensemble empirical mode decomposition for high frequency ECG noise reduction. Biomed. Tech. Biomed. Eng..

[36] Torres M.E., Colominas M.A., Schlotthauer G., Flandrin P. A complete ensemble empirical mode decomposition with adaptive noise. Proceedings of the IEEE International Conference on Acoustics, Speech and Signal Processing.

[37] Wang J., Li Z.C., Wang D.Y. (2014). A method for wavelet threshold de-noising of seismic data based on CEEMD. Geophys. Prospect. Pet..

[38] Huang W., Cai N., Xie W., Ye Q., Yang Z. (2015). ECG baseline wander correction based on ensemble empirical mode decomposition with complementary adaptive noise. J. Med. Imaging Health Inf..

[39] Jin J.J., Wang X., Wu X., Yang D. (2009). Translation-Invariant De-noising of Body Fluttering Signal Based on Improved Threshold Function. J. Northeast. Univ..

[40] Roonizi E.K., Sameni R. (2013). Morphological modeling of cardiac signals based on signal decomposition. Comput. Biol. Med..

